# NEDD4 Plays Roles in the Maintenance of Breast Cancer Stem Cell Characteristics

**DOI:** 10.3389/fonc.2020.01680

**Published:** 2020-09-02

**Authors:** Seon-Ae Jeon, Dong Wook Kim, Da-Bin Lee, Je-Yoel Cho

**Affiliations:** Department of Veterinary Biochemistry, BK21 Plus and Research Institute for Veterinary Science, School of Veterinary Medicine, Seoul National University, Seoul, South Korea

**Keywords:** TNBC, BCSC, NEDD4, LC–MS/MS, TMT labeling

## Abstract

Triple-negative breast cancer (TNBC) is the most aggressive type with poor prognosis among the breast cancers and has a high population of cancer stem cells (CSCs), which are the main target to cure and inhibit TNBC. In this study, we examined the role of neural precursor cell expressed developmentally downregulated protein 4 (NEDD4) in the proliferation, migration, and CSC characteristics of MDA-MB-231, a TNBC cell line. Interestingly, the Kaplan–Meier plotter showed that the survival rate of patients with a higher expression level of NEDD4 was significantly shorter than those of patients with a lower expression only in relatively aggressive and higher stage (grade 3) breast cancer patients. The knockdown of NEDD4 drastically decreased the proliferation, migration, and mammosphere formation in MDA-MB-231 cells. A proteomic analysis revealed the alteration of CSC-related proteins; notably, Myc targets stem cell-like signatures in siNEDD4-treated MDA-MB-231. An immunoassay also showed that the expression and the activity of breast CSC markers are decreased in NEDD4-deleted MDA-MB-231. Taken together, these results indicate that NEDD4 is involved in the maintenance of populations and characteristics of breast CSCs.

## Introduction

Breast cancer (BC) is the most common and malignant cancer in women (1) and classified into three subtypes depending on certain molecular biomarkers (2): estrogen receptor (ER)/progesterone receptor (PR)-positive, HER2-positive, and triple-negative breast cancers (TNBCs). The ER/PR-positive breast cancers account for more than 60% of breast cancer patients. The HER2-positive breast cancers comprise 10–20% of breast cancer patients and have a worse diagnosis than ER/PR-positive breast cancers (3). However, these two types of breast cancers are responsive to ER/PR and HER2-targeted therapy, which blocks their downstream signal activation. TNBC, which lacks ER/PR and HER2 on the surface of the cancer cells, occupies 15–20% of all breast cancer patients and has a worse outcome compared to ER/PR-positive and HER2-positive subtypes (4). Recently, a report suggested that TNBC can be classified into six subtypes (immunomodulatory, mesenchymal, mesenchymal stem-like, luminal androgen receptor, and two basal-like subtypes) by gene expression profile (5). Among the six subtypes of TNBCs, the stem cell-like group has a completely distinguished expression pattern of molecular signatures compared to ER/PR- and HER2-positive breast cancers.

The specific subset of cells termed cancer stem cells (CSCs) in both hematologic malignancies and solid tumors has the propensity for tumor initiation and recurrence. The population of these specific cells is very small in tumors, and they have the unique capacity for self-renewal, differentiation, invasion, and drug resistance (6, 7). It has been well described that breast cancer stem cells (BCSCs) also have specific properties in common with normal mammary stem cells and progenitor cells (8). Although there have been several proposed BCSC markers, the major three markers are a cluster of differentiation 44-positive (CD44^+^), CD24-negative (CD24^–^), and aldehyde dehydrogenase-positive 1 (ALDH1^+^) (9, 10). Interestingly, TNBCs have a relatively abundant BCSC population compared to other types of breast cancer, which makes these tumors more aggressive and drug resistant.

Neural precursor cell expressed developmentally downregulated protein 4 (NEDD4) is an E3 ubiquitin ligase which regulates target protein stability and cellular localization *via* proteosomal degradation (11). NEDD4 plays a critical role in diverse cellular functions in cancers, including tumor initiation, progression, migration, and resistance to anticancer therapies (12, 13). The expression of NEDD4 is frequently upregulated in several human cancers (13–15) and positively correlated with cell proliferation and survival *via* ubiquitination-mediated proteosomal degradation of tumor suppressors, such as PTEN (16, 17), and LATS (18, 19). In addition, NEDD4 exerts its oncogenic activity by stabilizing the mouse double minute 2 homolog that is also a ubiquitin ligase for p53, a tumor suppressor (20). Moreover, many other cancer-related signaling pathways are also regulated by NEDD4, such as pAKT, IGF1R, and NOTCH pathways (21–24). Given the role of NEDD4 in cancer, NEDD4 is considered to be a promising therapeutic target for the treatment of human malignancies (25). Although many signaling pathways regulated by NEDD4 in cancers have been discovered, the role of NEDD4 in CSCs remains elusive.

In this study, we analyzed the CSC-related proteome alteration by NEDD4 knockdown in CSC-abundant MDA-MB-231 cells by liquid chromatography–tandem mass spectrometry (LC–MS/MS) analysis. Then, we studied the effect of NEDD4 knockdown on the proliferation and the migration of MDA-MB-231 cells and the expression and the activity of the BCSC markers to reveal the role of NEDD4 in breast CSC maintenance.

## Materials and Methods

### Cell Culture and siRNA Transfection

MCF7, SKBR3, and MDA-MB-231 were purchased from Korea Cell Line Bank (Seoul, South Korea) and MDA-MB-436 was purchased from the American Type Culture Collection (Manassas, VA, United States). MCF7, MDA-MB-231, and MDA-MB-436 were cultured in Dulbecco’s modified Eagle medium with 10% fetal bovine serum (FBS), and SKBR3 was cultured in RPMI-1640 with 10% FBS in a humidified incubator at 37°C and 5% CO_2_. The transfection experiments of siRNAs were performed using Lipofectamine 3000 (Invitrogen, *Carlsbad*, CA, United States). NEDD4 siRNA was purchased from Bioneer (Daejeon, South Korea). The following siRNA oligonucleotides were used in the transfection studies: siNEDD4 (target sequence, 5′-ATGGAGTTGATTAGATTACAA-3′), 5′-GGAGUUGAUUAGAUUACAATT-3′ (sense strand), and 5′-UUGUAAUCUAAUCAACUCCAT-3′ (antisense strand).

### Quantitative PCR

Quantitative PCR was performed as previously reported (26). Briefly, total RNA was isolated from MDA-MB-231 using TRIzol reagent. Two micrograms of RNA was reverse-transcribed with an Omniscript reverse transcriptase kit for 1 h at 37°C. The differences in the mRNA expression of the target genes were determined by SYBR Green I fluorescence-based qRT-PCR. The mRNA level of each gene was normalized to *GAPDH*. The sequences of the primers are shown in Supplementary Table 1.

### Protein Isolation and Western Blotting

Protein isolation and western blotting were done as previously reported (26). Each protein band was normalized against β-actin. Anti-NEDD4 antibody (ab14592, Abcam; Cambridge, Cambridgeshire, United Kingdom) was used at 10,000:1 dilution, while anti-ALDH1A1 (ab134188) and anti-CD44 antibodies (ab157107, Abcam; Cambridge, Cambridgeshire, United Kingdom) as well as anti-β-actin antibody (A2228, Sigma Aldrich; St. Louis, MO, United States) were used at 1,000:1 dilution. The membrane was developed using a chemiluminescence detection system (ATTO Corporation, Tokyo, Japan).

### Filter-Aided Sample Preparation and Tandem Mass Tag Labeling

To prepare tryptic peptides for LC–MS/MS analysis, filter-aided sample preparation (FASP) was performed according to a previous report on siCONT- and siNEDD4-treated MDA-MB-231, respectively. Briefly, the cells were lysed by SDT lysis buffer [4% SDS, 100 mM Tris–HCl (pH 7.6), 0.1 M DTT]. Then, a total of 250 μg of proteins extracted by urea buffer (8 M urea in 0.1 M Tris–HCl, pH 8.5) was alkylated and digested by trypsin in FASP filters on the shaking incubator at 250 rpm overnight at 37°C. The digests were dried by centrifugation under vacuum. For a quantitative comparison, tandem mass tag (TMT) six-plex labeling was performed with 80 μg of tryptic peptides according to the manufacturer’s instruction (Thermofisher Scientific, Waltham, MA, United States).

### LC–MS/MS Analysis

Spectra raw data were acquired on Orbitrap Fusion Lumos (Thermofisher Scientific, Waltham, MA, United States) with EASY-nLC 1200 (Thermofisher Scientific, Waltham, MA, United States). An auto-sampler was used to load 10-μl aliquots of the peptide solutions into an EASY column—Acclaim PepMap^TM^ 100 of i.d. 75 μm, length 2 cm, and particle size of 3 μm (Thermofisher Scientific, Waltham, MA, United States). Then, the trapped peptides were separated on an EASY-Spray Column—C_18_ analytic-column of i.d. 75 μm, length 500 mm, and 2-μm particle size (100 Å from Thermofisher Scientific, Waltham, MA, United States). The mobile phases were composed of 100% water (buffer A) and 100% acetonitrile (buffer B), and each contained 0.1% formic acid. The LC gradient was initiated with 5% buffer B, increased to 8% buffer B over 1 min, 10% buffer B over 16 min, 40% buffer B over 79 min, and then maintained at 80% buffer B for 9 min and 2% buffer B for an additional 15 min at a flow rate of 300 nl/min. During the chromatographic separation, the Orbitrap Fusion Lumos was operated in a data-dependent acquisition mode. Survey full scans were acquired on mass range 400–1,600 m/z, maximum injection time of 100 ms, and automatic gain control (AGC) target 2e5 ions with resolution of 120,000 and analyzed using the Orbitrap. The MS/MS precursors were selected from top *n* intense ions in 3 s between the survey scans, which were fragmented by 37.5% higher collisional dissociation collision energy. The MS/MS data were acquired on a maximum injection time of 54 ms and AGC 5e4 ions with a resolution of 30,000 and analyzed using the Orbitrap. The previously fragmented precursors were excluded for 30 s.

### Protein Identification and Quantitative Data Analysis

Raw MS spectra were processed with MaxQuant software (version 1.5.8.3) (27) at default settings with unique peptide ≥ 2 and minimum number of amino acid ≥ 6. Identified peaks were searched against a database of human cancer from Uniprot^1^. The output files generated from Maxquant were subjected into Scaffold Q + software (version Scaffold_4.7.5, Proteome Software Inc., Portland, OR, United States) to TMT-labeled peptide and protein identifications. The peptide identifications were accepted if they could be established at greater than 5.0% probability to achieve a false discovery rate (FDR) of less than 1.0% by the Scaffold Local FDR algorithm. The protein identifications were accepted if they could be established at greater than 59.0% probability to achieve a FDR of less than 1.0% and contained at least two identified peptides. Protein probabilities were assigned by the Protein Prophet algorithm (28). The proteins that contained similar peptides and could not be differentiated based on MS/MS analysis alone were grouped to satisfy the principles of parsimony. Proteins sharing a significant peptide evidence were grouped into clusters. Normalization was performed iteratively (across samples and spectra) on intensities, as described in the statistical analysis of relative labeled mass spectrometry data from complex samples using ANOVA (29). Medians were used for averaging. The spectra data were log-transformed, pruned of those matched to multiple proteins and those missing a reference value, and weighted by an adaptive-intensity weighting algorithm.

### Wound Healing Assay

The cells treated with siCONT and siNEDD4 were, respectively, wounded with SPL Scarnd (SPL, Pocheon, Gyeonggido, South Korea), and the media were changed to freshly grown media. After 24 h, pictures were taken by microscopy (Revolve, ECHO, San Diego, CA, United States).

### Migration Assay

Cell migration ability was tested using 8.0-μm-pore polycarbonate-membrane-inserted transwell chambers (Corning, New York, NY, United States). The polycarbonate membrane of the upper chamber was coated with 0.1% gelatin, before the upper chambers with siCONT- and siNEDD4-treated cells (5 × 10^4^ for each chamber). After 3 h, the cells through the membrane were fixed with 4% paraformaldehyde for 5 min and stained with 1% crystal violet in 2% ethanol. Pictures of the stained cells were taken by microscopy (Revolve, ECHO, San Diego, CA, United States).

### Invasion Assay

Invasiveness ability was also tested using 8.0-μm-pore polycarbonate membrane-inserted transwell chambers (Corning, New York, NY, United States). The polycarbonate membrane of the upper chamber was coated with 0.3 mg/ml of matrigel, before the upper chambers with siCONT- and siNEDD4-treated cells (5 × 10^4^ for each chamber). After 24 h, cells through the membrane were fixed with 4% paraformaldehyde for 5 min and stained with 1% crystal violet in 2% ethanol. Pictures of the stained cells were taken by microscopy (Revolve, ECHO, San Diego, CA, United States).

### ALDEFLUOR Assay

For the evaluation of ALDH1A1 activity, an ALDEFLUOR kit (STEMCELL Technologies, Vancouver, BC, Canada) was used according to the manufacturer’s instructions. Briefly, siCONT/NEDD4-treated cells were cultured in growth media supplemented with 10 μl ALDEFLUOR reagent for 30 min, and then green fluorescence was detected by microscopy (Revolve, ECHO, San Diego, CA, United States).

### Statistical Analysis

The data represent the means ± SEM. GraphPad Prism software (version 7.01, GraphPad software, Inc., CA, United States) was used for statistical analyses. The statistical significance of the results was assessed using Student’s *t* test (two-tailed). For all experiments, significance was defined as ^∗^*P* < 0.05, ^∗∗^*P* < 0.01, and ^∗∗∗^*P* < 0.001.

## Results

### The Relationship Between the Expression of NEDD4 and the Survival Rate of Breast Cancer Patients

**FIGURE 1:**
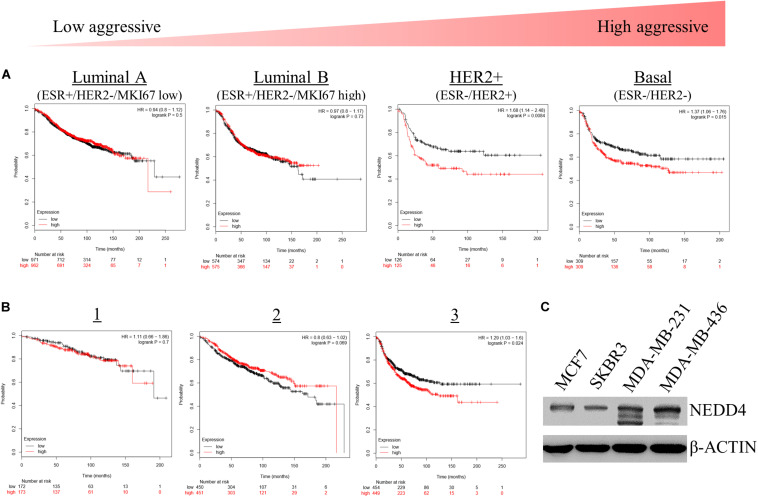
The expression level of NEDD4 is negatively correlated with the survival rate in aggressive breast cancer patients. **(A,B)** The Kaplan–Meier survival curve showing the correlation between the level of NEDD4 expression and the aggressiveness and stage in breast cancer patients. **(C)** The expression of NEDD4 was confirmed in human breast cancer cell lines by western blot with an anti-NEDD4 antibody. β-actin was used as a loading control.

We assessed the effects of NEDD4 level on the survival rate of breast cancer patients. For that, we used the Kaplan–Meier (KM) plotter, which is a web-based tool that shows the effect of 54,000 genes on the survival rate of cancer patients^2^. The KM plotter revealed that the survival rate of patients with a high expression of NEDD4 was shorter than that of patients with a low expression of NEDD4 in HER2-positive (HER2^+^) and TNBC, which have been known as relatively aggressive breast cancers (Figure 1A). In addition, the statistically significant positive relationship appeared distinctively in the advanced stage of breast cancer as opposed to the early stage (Figure 1B). A western blot analysis showed that the expression level of NEDD4 was relatively higher in MDA-MB-231 (TNBC) and MDA-MB-436 (TNBC) than in MCF7 (luminal type) and SK-BR-3 (HER2 + type). As a result, the higher the expression of NEDD4, the more aggressive and the lower the survival rate of the breast cancer.

### NEDD4 Knockdown Inhibited the Proliferation and the Migration of MDA-MB-231

**FIGURE 2:**
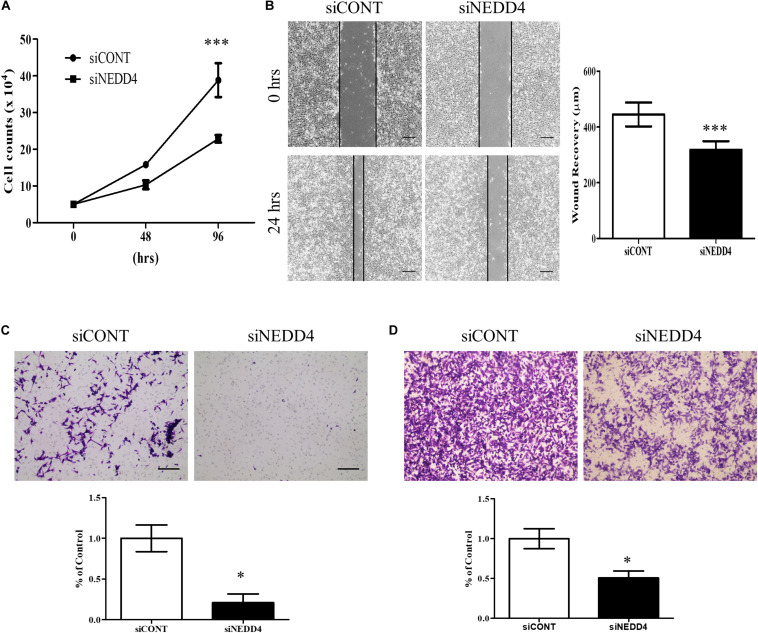
Cell proliferation and migration are decreased in NEDD4-deleted MDA-MB-231. **(A)** The cell proliferation rate was confirmed by cell counting at 48 and 96 h after transfection of control siRNA (siCONT) and siNEDD4 into MDA-MB-231 (*n* = 3). **(B)** The wound healing assay was performed in siCONT- and siNEDD4-treated MDA-MB-231. At 24 h after wounding, the images were captured to measure the distance that was filled through cell migration and proliferation. The representative images are shown. The average value is shown in the graph (*n* = 3). Scale bar, 10 μm. **(C)** Cell migration assay was performed with Transwell cell culture chambers in siCONT- and siNEDD4-treated MDA-MB-231. Scale bar, 10 μm. **(D)** The invasiveness capacity was confirmed with matrigel-coated transwell chambers in siCONT- and siNEDD4-treated MDA-MB-231. The representative images are shown (*n* = 3) (*P < 0.05 and ***P < 0.001).

For further experiments, the MDA-MB-231 cell line was selected due to its high expression of NEDD4 (Figure 1C) and its TNBC properties, including being positive for BCSC markers (30). Proliferation and migration were confirmed in siCONT- and siNEDD4-treated MDA-MB-231. The proliferation was significantly decreased in siNEDD4-treated MDA-MB-231 (Figure 2A). The migration was also confirmed to be decreased in knockdown cells by both wound healing assay (Figure 2B) and transwell system (Figure 2C). The invasiveness was also decreased in siNEDD4-treated MDA-MB-231 than in the control (Figure 2D). To make the results more convincing, proliferation, migration, and invasion assays were also conducted in siCONT- and siNEDD4-treated MDA-MB-436, one of the TNBC cell lines. The proliferation was confirmed to be decreased by NEDD4 knockdown by cell counting (Supplementary Figure 1A). The migration and the invasion capacity were also decreased in siNEDD4-treated MDA-MB-436 (Supplementary Figures 1B,C). Taken together, MDA-MB-231 cell viability, in the form of proliferation and migration, is affected by NEDD4.

### Identification and Functional Characterization of Differentially Expressed Proteins in NEDD4-Knocked-Down MDA-MB-231 by LC–MS/MS

**FIGURE 3:**
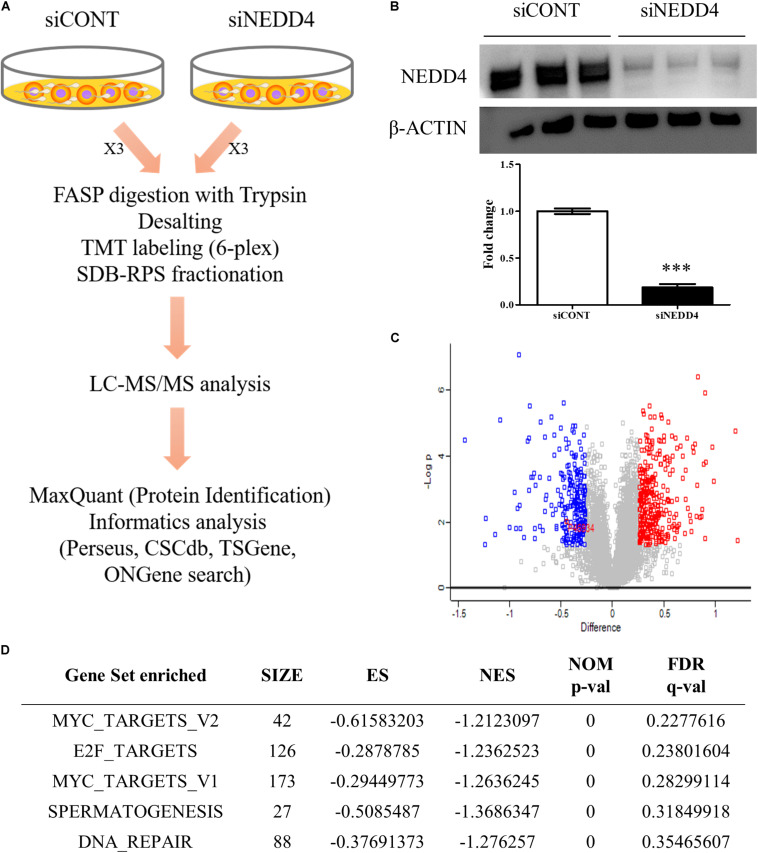
Identification of differentially expressed proteins (DEPs) by liquid chromatography–tandem mass spectrometry (LC–MS/MS) analysis in NEDD4-deleted MDA-MB-231. **(A)** Schematic diagram showing the overall workflow for proteomics analysis in siCONT- and siNEDD4-treated MDA-MB-231. The peptides obtained by trypsin digestion were labeled by tandem mass tag six-plex reagents. Then, identification and functional analysis of DEPs were performed by LC–MS/MS and bioinformatics tools. **(B)** The efficiency of NEDD4 knockdown by transfection of siNEDD4 into MDA-MB-231 was confirmed by western blot with an anti-NEDD4 antibody. β-actin was used as a loading control. **(C)** The volcano plot obtained from “Perseus” shows differentially expressed proteins in siNEDD4-transfected MDA-MB-231 compared to siCONT-transfected ones. Proteins in red or blue were considered as significantly up- or downregulated by using Perseus software. **(D)** Gene set enrichment analysis showing the gene sets enriched in downregulated proteins by NEDD4 knockdown (***P < 0.001).

To identify the altered proteomes by NEDD4 deletion in MDA-MB-231, LC–MS/MS analysis was conducted. Proteins isolated from siCONT- and siNEDD4-treated MDA-MB-231 were digested using the FASP method and labeled by TMT six-plex reagent. Following this, the combined samples were fractionated to three by styrene divinylbenzene–reversed-phase sulfonate and analyzed by LC–MS/MS (Figure 3A). The western blot analysis using anti-NEDD4 antibody showed that the expression of NEDD4 was reduced by more than 90% by transfection of siNEDD4 (Figure 3B). The volcano plot showed that 333 proteins were increased and 252 proteins were decreased in siNEDD4-treated MDA-MB-231 as opposed to those in siCONT-treated cells (Figure 3C and Supplementary Tables 2, 3). Accordingly, the reduced expression of NEDD4 by siNEDD4 treatment was also confirmed by LC–MS/MS.

To further categorize and visualize the function and the pathway enrichment of differentially expressed proteins, gene set enrichment analysis was performed. A previous report determined that poorly differentiated aggressive human breast cancers showed embryonic stem cell-like signatures, including 13 gene sets (31). Interestingly, compared to the control, the NEDD4-knockdowned MDA-MB-231 cells had a lower expression of genes enriched by “MYC_TARGETS_V2 (*p*-value < 0.05, FDR *q*-value = 0.23)” and “MYC_TARGETS_V1 (*p*-value < 0.05, FDR *q*-value = 0.28),” which are known as embryonic stem cell-like gene signatures (Figure 3D).

**FIGURE 4:**
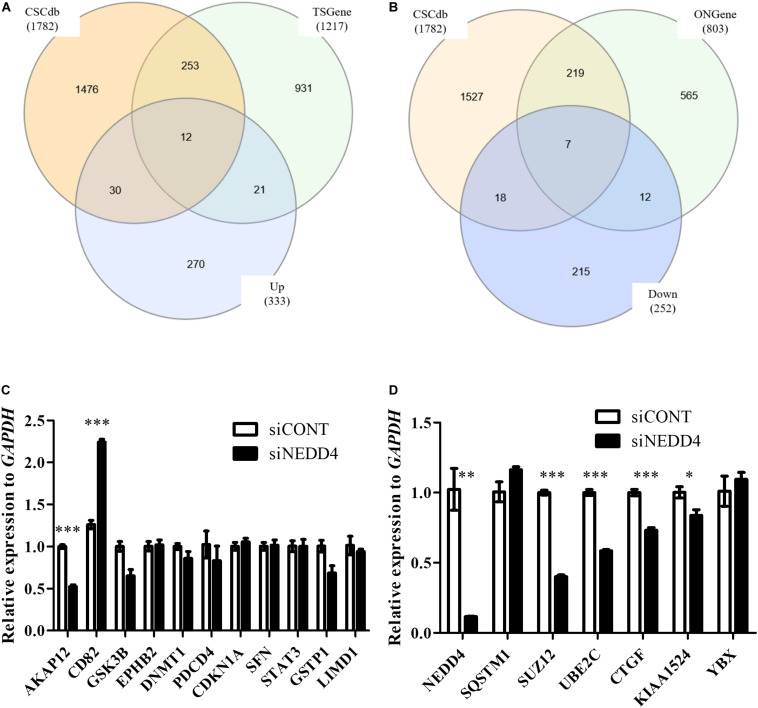
A proteomic analysis reveals that the expression of cancer stem cell (CSC)-related proteins is altered by NEDD4 deletion. **(A)** Venn diagram showing the overlaps of upregulated proteins with CSC-related (CSCdb) and tumor suppressors (TSGene). **(B)** Venn diagram showing the overlaps of downregulated proteins with CSC-related (CSCdb) and oncogenes (ONGene). **(C)** The mRNA expression of CSC-related and increased proteins by NEDD4 knockdown was confirmed by qPCR in siCONT/NEDD4-treated MDA-MB-231, respectively. **(D)** The mRNA expression of CSC-related and decreased proteins by NEDD4 knockdown was confirmed by qPCR in siCONT/NEDD4-treated MDA-MB-231, respectively (*P < 0.05; **P < 0.01, and ***P < 0.001).

Next, to distinguish the CSC-related proteins among those differentially expressed by NEDD4 knockdown, up/downregulated proteins were matched with CSC-related proteins excerpted from CSCdb (32). A number of studies have shown that NEDD4 is an oncoprotein that catalyzes the ubiquitination and the degradation of target proteins that are commonly known as tumor suppressors, such as PTEN and LATS (16, 18). We thus hypothesized that upregulated proteins by NEDD4 knockdown include tumor suppressors and potential candidates for ubiquitination targets. In addition, downregulated proteins are regarded as oncoproteins. Therefore, known tumor suppressors (TSGene) (33), and oncogenes (ONGene) (34) were selected from the 333 upregulated and 252 downregulated proteins, respectively. Finally, 12 upregulated proteins, such as AKAP12, CD82, GSK3B, EPHB2, DNMT1, PDCD4, PEBP1, CDKN1A, SFN, STAT3, GSTP1, and LIMD1, were matched with both CSC-related and tumor suppressors (Figure 4A and Table 1), and seven downregulated proteins, such as SQSTM1, SUZ12, UBE2C, EZH2, CTGF, KIAA1524, and YBX1, were matched with both CSC-related and oncoproteins (Figure 4B and Table 2). The mRNA expression of some up/downregulated proteins was confirmed by qPCR. In the condition that generally about 30–40% mRNA expression correlates with protein expression, our qPCR data showing that the mRNA expression of CD82 increased (Figure 4C) and SUZ12, UBE2C, CTGF, and KIAA1524 decreased (Figure 4D) in siNEDD4-treated MAD-MB-231 cells were validated to proteomic data.

**TABLE 1:** Cancer stem cell-related and upregulated proteins by NEDD4 knockdown in MDA-MB-231.

**Gene name**	**Protein name**	**Fold change**	***p*-value**
AKAP12	A-kinase anchor protein 12	1.66	***
CD82	CD82 antigen	1.61	***
GSK3B	Glycogen synthase kinase-3 beta	1.52	*
EPHB2	Ephrin type-B receptor 2	1.5	***
DNMT1	DNA (cytosine-5)-methyltransferase 1	1.48	***
PDCD4	Programmed cell death protein 4	1.42	***
PEBP1	Phosphatidylethanolamine-binding protein 1; hippocampal cholinergic neurostimulating peptide	1.35	***
CDKN1A	Cyclin-dependent kinase inhibitor 1	1.31	**
SFN	14-3-3 protein sigma	1.24	**
STAT3	Signal transducer and activator of transcription 3	1.23	**
GSTP1	Glutathione S-transferase P	1.22	***
LIMD1	LIM domain-containing protein 1	1.2	**

*P < 0.05; **P < 0.01, and ***P < 0.001.

**TABLE 2:** Cancer stem cell-related and downregulated proteins by NEDD4 knockdown in MDA-MB-231.

**Gene name**	**Protein name**	**Fold change**	***p*-value**
SQSTM1	Sequestosome-1	0.73	***
SUZ12	Polycomb protein SUZ12	0.76	***
UBE2C	Ubiquitin-conjugating enzyme E2 C	0.77	*
EZH2	Histone-lysine N-methyltransferase EZH2	0.81	***
CTGF	Connective tissue growth factor	0.82	*
KIAA1524	Protein CIP2A	0.83	***
YBX1	Nuclease-sensitive element-binding protein 1	0.83	**

*P < 0.05; **P < 0.01, and ***P < 0.001.

### The Properties of BCSC Were Decreased by NEDD4 Knockdown

**FIGURE 5:**
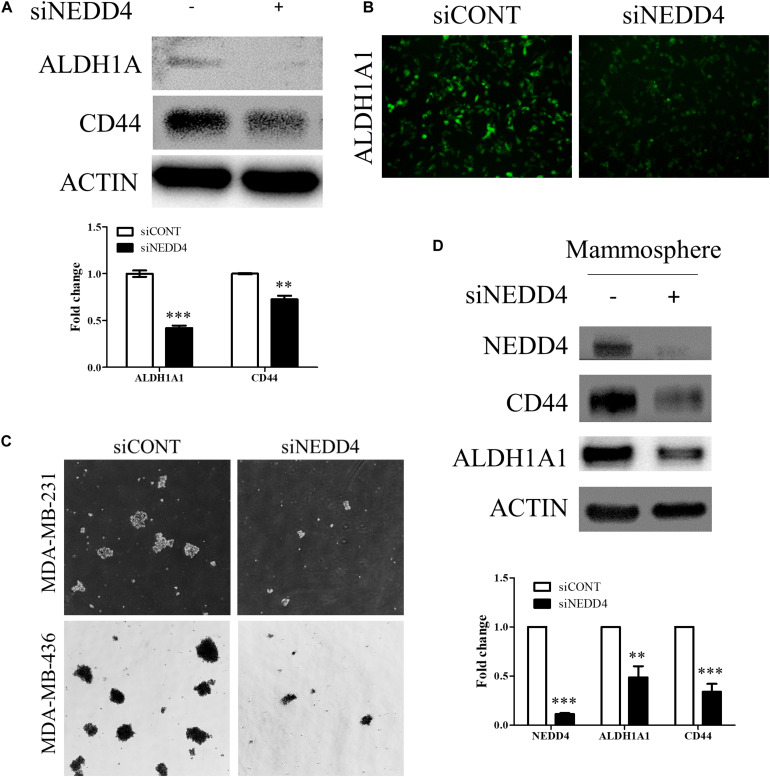
The expression and the activity of breast cancer stem cell (BCSC) markers were decreased in NEDD4-deleted MDA-MB-231. **(A)** The expression of BCSC markers including ALDH1A1 and CD44 was confirmed by western blot in siCONT- and siNEDD4-treated MDA-MB-231. β-actin was used as a loading control. **(B)** The activity of ALDH1A1 was confirmed by the ALDEFLUOR kit system in siCONT/NEDD4-treated MDA-MB-231 cells. The representative images are shown. The average value is shown in the graph (*n* = 3). **(C)** siCONT/NEDD4-treated MDA-MB-231 cells were induced to mammosphere formation for 2 weeks. The representative images are shown (*n* = 3). **(D)** The lysates harvested from the mammosphere formation were subjected to western blot using the indicated antibodies. β-actin was used as a loading control. The representative images are shown. The average value is shown in the graph (*n* = 3) (**P < 0.01 and ***P < 0.001).

We also investigated whether the expression and the activity of BCSC markers are altered by NEDD4 knockdown. The expression of ALDH1A1 and CD44 was reduced in siNEDD4-treated MDA-MB-231 cells (Figure 5A). The activity of ALDH1A1 was also confirmed with the ALDEFLUOR assay. NEDD4-knocked-down MDA-MB-231 cells resulted in a much weaker green intensity, which indicates low ALDH1A1 activity (Figure 5B). These observations led us to test whether NEDD4 knockdown suppresses mammosphere formation, which is also a stem cell-like phenotype. To this end, siCONT- and NEDD4-treated MDA-MB-231 were cultured in mammosphere media for 2 weeks. Expectedly, the knockdown of NEDD4 inhibited big and round mammosphere formation (Figure 5C). Also, the expression of BCSC markers including ALDH1A1 and CD44 was decreased by NEDD4 knockdown (Figure 5D). Taken together, NEDD4 is required for the maintenance of breast CSC characteristics.

## Discussion

**FIGURE 6:**
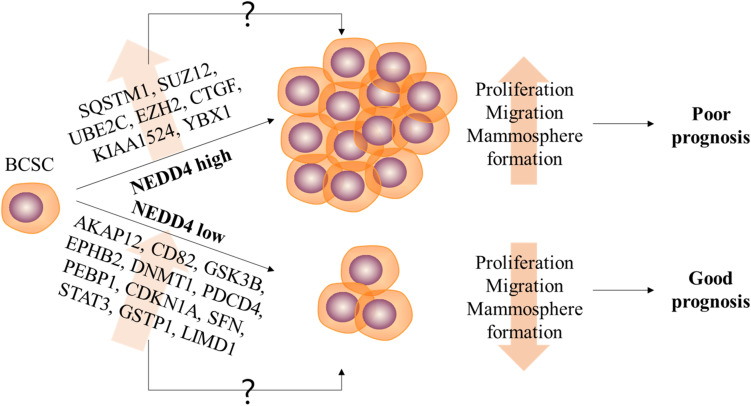
The role of NEDD4 in the regulation of breast cancer stem cell (BCSC) properties in MDA-MB-231. The schematic model shows that NEDD4 is required for proliferation, migration, and stemness maintenance in BCSCs. The breast cancer patients with a high expression of NEDD4 in BCSCs may show a shorter survival period and a poor prognosis due to the increased proliferation, migration, and stemness of BCSCs, while the patients with a low level of NEDD4 in BCSCs may live longer and have a better prognosis. Although detailed researches were needed, our proteomics analysis revealed that the differentially expressed proteins by NEDD4 knockdown were related to BCSCs.

The expression and the molecular function of NEDD4 have frequently been studied in various human cancers. However, only a few studies have reported on the role of NEDD4 in CSCs. Targeting CSCs in cancer treatment is very important because of their tumor-initiating and metastatic capacity and since non-CSCs enduring chemotherapy treatment can acquire CSC features leading to poor prognosis (7). In this study, we report the reduced properties of CSCs and CSC-related DEPs by NEDD4 knockdown. The proliferation and the migration of MDA-MB-231 are decreased, and the expression of BCSC markers and the ability to form mammosphere are also significantly reduced by NEDD4 knockdown (Figure 6).

Numerous studies have suggested a lot of downstream substrates, and upstream regulators of NEDD4 are known to be involved in carcinogenesis (35, 36). However, only a few studies have investigated the role of NEDD4 in breast cancer (BC) and BCSC. One such study showed that the expression of NEDD4 was elevated, and it promoted the growth of BC (37), yet the BC-type dependency has not been considered in studying the function of NEDD4. In this study, we realized that breast cancer patients with highly expressing NEDD4 show significantly reduced survival rates, specifically in poorly differentiated breast cancer types which are ER-negative breast cancer types, including HER2^+^ and TNBC (Figure 1A). Although the expression and the role of ER in BCSCs remain under debate, several studies have shown that the expression level of ER was very low or none in CD44^+^/CD24^–^/ALDH^+^ CSCs (38, 39). Moreover, ALDH^–^ BCSCs were associated with ER-negativity, HER2-positivity, high histological grade, and poor prognosis (40). Furthermore, breast cancers lacking the expression of ER showed embryonic stem cell-like gene set enrichment (31).

In this study, we focused on the role of NEDD4 in BCSCs, which may account for a shorter survival rate in the patients. Several studies show that the number of BCSCs was increased by stimulation with estradiol in spite of ER-negativity in BCSCs. In ER-negative BCSCs, G-protein coupled receptor (GPCR) 30 could mediate estrogen action. The GPCR-mediated Hippo-TAZ pathway was also required to activate the self-renewal capacity of non-CSCs (41, 42). The increased TAZ activation was detected in poorly differentiated breast cancers, and it also enhanced the metastatic features. Other reports indicate that breast cancers highly expressing type 1 tyrosine kinase-like orphan receptor also have stem cell-like features through activation of Rho-GTPase and the Hippo-YAP/TAZ signaling pathway (43). NEDD4 has been known as a regulator of the Hippo signaling pathway through ubiquitination and degradation of LATS kinase (18, 19, 44). Although more detailed experiments and analysis are needed, NEDD4 might regulate the stem cell-like characteristics in BCSCs by activating the GPCR-mediated Hippo-YAP/TAZ pathway.

A previous report exhibited that ES gene set signatures including ES exp1, ES exp2, Nanog targets, Oct4 targets, Sox2 targets, NOS targets, NOS TFs, Myc target1, and Myc target2 were overexpressed and gene sets including Suz12 targets, Eed targets, H3K27 bound, and PRC3 targets were underexpressed in ER-negative and high grade 3 breast cancer patients (31). Our TMT-based high-throughput proteomics analysis revealed 585 differentially expressed proteins (DEPs) in siNEDD4-treated MDA-MB-231, and these DEPs are functionally associated with the maintenance of BCSC properties. The two gene sets including Myc_target1 and Myc_target2 are especially enriched in downregulated proteins following NEDD4 knockdown (Figure 3D), indicating that the ESC-like features in ER-negative and highly aggressive breast cancers are lost with NEDD4 knockdown.

Our LC–MS/MS analysis also revealed that the expression of both EZH2 and SUZ12 is significantly decreased (Table 2), and the mRNA level of SUZ12 is also reduced in siNEDD4-treated MDA-MB-231 (Figure 4D). EZH2 and SUZ12 both belong to polycomb-repressive complex 2 (PRC2), which is necessary for the maintenance of stem cell pluripotency and the inhibition of differentiation by activation of histone H3 lysine 27 trimethylation (H3K27me3) that represses the transcription of the differentiation genes (45). It also interacted with BRCA1, a tumor suppressor, in both ES cells and breast cancers and acted as a negative modulator of PRC2. The loss of BRCA1 led to the activation of PRC2, inhibition of cell differentiation, and induction of more aggressive breast cancers (46). PRC2 is composed of four core subunits: EZH2, SUZ12, EED, and RbAp46/48. The expression of EZH2 and SUZ12 was increased in several types of cancers such as prostate, ovarian, and breast cancers, and it initiated tumorigenesis and activated anti-drug responses through preventing the expression of differentiation-related genes and promoting stem cell-like phenotypes (47–50). Thus, our results suggest that NEDD4 is involved in the PRC2 complex which inhibits the transcription of differentiation genes.

In conclusion, our results indicate that the systems which regulate the maintenance of stemness are negatively regulated by NEDD4 knockdown, which affects the formation of the mammosphere and the expression of BCSC markers such as ALDH1A1 and CD44. We believe that such discoveries will help to provide much insight into the development of novel therapeutics and drugs targeting NEDD4 in a variety of CSCs.

## Data Availability Statement

The original contributions presented in the study are publicly available. This data can be found here: http://proteomecentral.proteomexchange.org/cgi/GetDataset, Accession: PXD020585.

## Author Contributions

S-AJ, DK, and J-YC designed the research, analyzed the data, developed the methodologies, and reviewed and revised the manuscript. S-AJ performed the experiments. S-AJ, DK, and D-BL conducted the bioinformatics analysis. D-BL operated the LC-MS/MS analysis. S-AJ wrote the manuscript draft. J-YC supervised this study. All the authors read and approved the final manuscript.

## Conflict of Interest

The authors declare that the research was conducted in the absence of any commercial or financial relationships that could be construed as a potential conflict of interest.

## Footnotes

1 https://www.uniprot.org/

2 https://kmplot.com/analysis/index.php?p=service&cancer=breast
