# Supplementary material for: NEDD4 Plays Roles in the Maintenance of Breast Cancer Stem Cell Characteristics
Source: Front Oncol. 2020 Sep 2;10:1680. doi: 10.3389/fonc.2020.01680 (PMC7509455; doi:10.3389/fonc.2020.01680)

**Supplementary figure 1. Cell proliferation and migration are decreased in NEDD4-deleted MDA-MB-436. (A)** Cell proliferation rate was confirmed by cell counting at 48 and 96 hours after transfection of control siRNA (siCONT) and siNEDD4 into MDA-MB-436 (n=3). **(B)** Cell migration assay was performed with Transwell cell culture chambers in siCONT and siNEDD4-treated MDA-MB-436. The representative images are shown (n=3) **(C)** The invasiveness capacity was confirmed with matrigel-coated transwell chambers in siCONT and siNEDD4-treated MDA-MB-436. The representative images are shown (n=3).


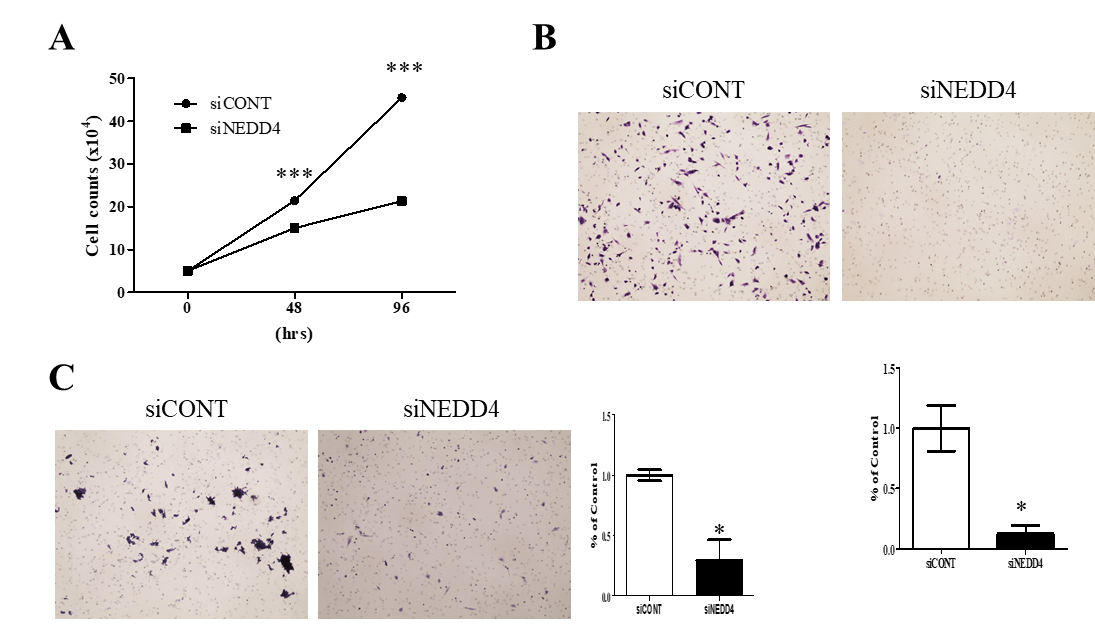

Supplement: Supplementary file 1 [file Data_Sheet_1.docx]
